# Catalytically inactive DNA ligase IV promotes DNA repair in living cells

**DOI:** 10.1093/nar/gkac913

**Published:** 2022-10-20

**Authors:** Noah J Goff, Manon Brenière, Christopher J Buehl, Abinadabe J de Melo, Hana Huskova, Takashi Ochi, Tom L Blundell, Weifeng Mao, Kefei Yu, Mauro Modesti, Katheryn Meek

**Affiliations:** College of Veterinary Medicine, Michigan State University, East Lansing, MI 48824, USA; Department of Microbiology & Molecular Genetics, Michigan State University, East Lansing, MI 48824, USA; Department of Pathobiology & Diagnostic Investigation, Michigan State University, East Lansing, MI 48824, USA; Centre de Recherche en Cancérologie de Marseille, CNRS UMR7258, Inserm U1068, Institut Paoli-Calmettes, Aix-Marseille Universiteé, Marseille, France; College of Veterinary Medicine, Michigan State University, East Lansing, MI 48824, USA; Department of Microbiology & Molecular Genetics, Michigan State University, East Lansing, MI 48824, USA; Department of Pathobiology & Diagnostic Investigation, Michigan State University, East Lansing, MI 48824, USA; Centre de Recherche en Cancérologie de Marseille, CNRS UMR7258, Inserm U1068, Institut Paoli-Calmettes, Aix-Marseille Universiteé, Marseille, France; Centre de Recherche en Cancérologie de Marseille, CNRS UMR7258, Inserm U1068, Institut Paoli-Calmettes, Aix-Marseille Universiteé, Marseille, France; The Astbury Centre for Structural Molecular Biology, School of Molecular and Cellular Biology, Faculty of Biological Sciences, University of Leeds, Leeds LS2 9TJ, UK; Department of Biochemistry, University of Cambridge, Cambridge CB2 1GA, UK; College of Human Medicine, Michigan State University, East Lansing, MI 48824, USA; Department of Microbiology & Molecular Genetics, Michigan State University, East Lansing, MI 48824, USA; College of Human Medicine, Michigan State University, East Lansing, MI 48824, USA; Department of Microbiology & Molecular Genetics, Michigan State University, East Lansing, MI 48824, USA; Centre de Recherche en Cancérologie de Marseille, CNRS UMR7258, Inserm U1068, Institut Paoli-Calmettes, Aix-Marseille Universiteé, Marseille, France; College of Veterinary Medicine, Michigan State University, East Lansing, MI 48824, USA; Department of Microbiology & Molecular Genetics, Michigan State University, East Lansing, MI 48824, USA; Department of Pathobiology & Diagnostic Investigation, Michigan State University, East Lansing, MI 48824, USA

## Abstract

DNA double strand breaks (DSBs) are induced by external genotoxic agents (ionizing radiation or genotoxins) or by internal processes (recombination intermediates in lymphocytes or by replication errors). The DNA ends induced by these genotoxic processes are often not ligatable, requiring potentially mutagenic end-processing to render ends compatible for ligation by non-homologous end-joining (NHEJ). Using single molecule approaches, Loparo *et al.* propose that NHEJ fidelity can be maintained by restricting end-processing to a ligation competent short-range NHEJ complex that ‘maximizes the fidelity of DNA repair’. These *in vitro* studies show that although this short-range NHEJ complex requires DNA ligase IV (Lig4), its catalytic activity is dispensable. Here using cellular models, we show that inactive Lig4 robustly promotes DNA repair in living cells. Compared to repair products from wild-type cells, those isolated from cells with inactive Lig4 show a somewhat increased fraction that utilize micro-homology (MH) at the joining site consistent with alternative end-joining (a-EJ). But unlike a-EJ in the absence of NHEJ, a large percentage of joints isolated from cells with inactive Lig4 occur with no MH – thus, clearly distinct from a-EJ. Finally, biochemical assays demonstrate that the inactive Lig4 complex promotes the activity of DNA ligase III (Lig3).

## INTRODUCTION

A growing consensus is emerging that DNA double-strand break (DSB) repair by the non-homologous end-joining (NHEJ) pathway proceeds through distinct steps (1–8). Loparo *et al.* have shown that a long-range synaptic complex that positions the DNA ends ∼115 angstroms apart is dependent on the catalytic subunit of the DNA dependent protein kinase (DNA-PKcs, DNA-PK) and its regulatory subunit the DNA end-binding factor Ku. Transition of these long-range complexes to short-range synaptic complexes requires the catalytic activity of DNA-PK and the NHEJ ligase complex including XRCC4, XLF, and DNA ligase IV (Lig4). These *in vitro s*tudies establish that whereas the long-range complex effectively blocks DNA end-processing, the short-range complex facilitates both end-processing and ligation (3,9). These studies are in good agreement from cellular studies from Ramsden and colleagues who propose that the ligase complex helps to limit end-processing to promote more error-free repair (10). Of note, in these *in vitro* studies, the catalytic activity of Lig4 is not required to either promote formation of the short-range complex or to facilitate end-processing that occurs in the complex. This result is consistent with previous studies proposing various structural roles for the Lig4 complex including promotion of end-processing (11), promotion of end-synapsis (12–14), and notably a report from Chiruvella *et al.* who show that in yeast, a catalytically inactive Lig4 complex increases chromosomal end-joining (15). Support for this emerging two-step model of NHEJ has been bolstered by recent cryo-EM studies that precisely delineate potential long-range and short-range NHEJ complexes (4–6).

In these previous studies, although end-processing mediated by the X family polymerases polλ and polμ, as well as TDP1, and PNKP was clearly dependent on the short-range complex, end-processing by the Artemis nuclease was not (9). Artemis functions exclusively with DNA-PKcs; its role in facilitating opening of the hairpin DNA termini associated with VDJ recombination [the process that provides for the generation of a diverse repertoire of antibodies and T cell receptors] has been well-defined (16–18). However, Artemis also functions to repair a subset of DSBs with non-ligatable DNA ends (19). Work from our laboratory also strongly suggests that Artemis hairpin opening is not restricted to the short-range complex (20). Briefly, using episomal end-joining assays, we found that cells which lack the NHEJ ligase complex robustly open hairpin termini. These data are consistent with experiments in developing mouse lymphocytes from NHEJ deficient mice. In these studies, opened hairpin coding joints are observed in mice with defects in the ligase complex (21), but not in mice with defects in either DNA-PK or Artemis (22,23); but, coding end-joining is dramatically impaired in mice with any of these defects in NHEJ (22). Finally, these results are completely consistent with recent structural studies that demonstrate that DNA-PK and Artemis function in a complex that does not require the Lig4 complex that is apparently requisite for other end processing activities (24).

It has been known for decades that NHEJ defective cell lines robustly repair DSBs on episomes using the alternative non-homologous end-joining pathway (a-EJ) (25,26). However, our recent study demonstrates that cells lacking the XRCC4/Lig4 complex are similarly defective in joining the opened hairpins as are cells lacking either DNA-PKcs or Artemis where the hairpins remain sealed (20). This suggests that the DNA-PK/Artemis complex that facilitates hairpin opening also, via some undefined mechanism, shields the opened hairpin ends from a-EJ; this ‘undefined mechanism’ would be consistent with the function of the long-range complex (blocking end-processing and ligation) proposed by Loparo *et al.* (9).

Here we explore the potential basis for Lig4’s non-catalytic role in NHEJ. To test whether the presence of the ligase complex (but not its ligase activity) is sufficient to promote end-processing in living cells, we generated (via CRISPR/Cas9 strategies) both Lig4 deficient and enzymatically inactive Lig4 mutant cell strains. We find that whereas Lig4 deficient cells are similarly impaired in joining DSBs with hairpin termini as are other NHEJ defective cells, cells expressing inactive Lig4 are remarkably proficient in rejoining these DSBs. We conclude that the DNA-PK complex and perhaps the long-range synaptic complex protects DSBs from other DNA ligases and end-processing factors (except for Artemis) in living cells. Moreover, our cellular experiments demonstrate that a catalytically inactive Lig4 complex can efficiently promote end-joining in living cells, consistent with the model that NHEJ-mediated end-processing is limited to a short-range synaptic complex. It follows that either Lig3 or Lig1 must be capable of facilitating ligation of DNA ends processed by NHEJ’s short-range complexes. Thus, we used *in vitro* assays and establish that the catalytically inactive Lig4 complex robustly promotes the activity of Lig3 but not Lig1. Finally, repaired DSBs recovered from cells expressing catalytically inactive Lig4 have increased utilization of short sequence micro-homologies (MH) at the joining site, a well-established characteristic of a-EJ (27).

## MATERIALS AND METHODS

### Cell culture, genome editing, and survival assays

293T, U2OS and HCT116 cells were cultured in Dulbecco's Modified Eagle Medium (Life Technologies) supplemented with 10% fetal bovine serum (Atlanta Biologicals, GA), 2 mM L-glutamine, 0.1 mM non-essential amino acids, 1 mM sodium pyruvate, 100 U/ml penicillin, 100 μg/ml streptomycin and 25 ng/ml Gibco Amphotericin B (Life Technologies). CH12F3 cells were cultured in RPMI 1640 medium supplemented with 10% (vol/vol) FBS and 50 μM β-mercaptoethanol.

Cas9-targeted gene disruption was performed using methods similar to those reported by Mali *et al.* (28). Briefly, duplex oligonucleotides (Integrated DNA Technologies) generating a gRNA specific for a PAM site targeting the K273 codon were cloned into px459 (Addgene #62988). Cells were transfected with 2 μg plasmid and a 120 nucleotide oligonucleotide encoding a K273A mutation as well as a silent mutation generating a novel restriction site (HhaI) (see Supplementary Table S1). 48 hours after transfection, cells were replated at cloning densities in media containing puromycin (1μg/ml). Puromycin was removed after 72 hours. Isolated clones were selected, and DNA isolated with DNAzol (Sigma) according to the manufacturer's protocol. Restriction digestion of PCR products was utilized to detect the K273A mutation. Western blotting was used to confirm expression and genotypes were confirmed by Sanger sequencing or Amplicon sequencing (Genewiz) as depicted in Supplementary Table S1.

MTT staining was performed to assess cell viability for both 293T cells and CH12F3. Cells were plated into 24-well plates, containing medium with varying concentrations of calicheamicin. After 5–7 days of calicheamicin treatment, cells were treated with 1 mg/ml MTT (Sigma) solution for 1 h. Medium containing MTT was then removed and formazan crystals thus produced were solubilized in DMSO. Absorbance was read at 570 nm to determine relative survival.

### Episomal end-joining assays

The fluorescent VDJ coding, signal, and alt-VDJ substrates have been described (20,29,30). The Fill-in substrate was generated by synthesizing (Integrated DNA Technologies) a fragment encoding crimson with the addition of restriction endonuclease sites Eco53KI, PspOMI, and Apa1 that disrupt the Crimson open-reading frame. Cleavage with these restriction enzymes generate blunt and over-hanged ends as described in Figure 7A. The Crimson fragment included NheI and BamHI which were used to subclone this fragment between the promoter and GFP open reading frame in the substrate plasmids. Briefly, extrachromosomal fluorescent joining assays were performed on cells plated at 20–40% confluency into 24-well plates in complete medium. Cells were transfected with 0.125 μg substrate, and either 0.25 I-Sce1, TelN, or RAG 1 + 2 expression plasmids per well using polyethylenimine (PEI, 1 μg/ml, Polysciences) at 2 μl/1 μg DNA. Cells were harvested 72 hours after transfection and analyzed for GFP and RFP expression by flow cytometry. The percentage of recombination was calculated as the percentage of live cells expressing GFP divided by the percentage expressing RFP. Data presented represents at least three independent experiments, which each includes triplicate transfections.

In Figure 7, the coding joint substrate was modified so that the coding flanks included only A or T sequences; in addition, the 23RSS was inverted to provide for inversional joining which facilitates PCR amplification of coding joints. Transfected plasmids were isolated by alkaline lysates 72 h after transfection. Coding joints from each transfection were PCR amplified and analyzed by electrophoresis and amplicon sequencing by Genewiz.

### Class switch recombination assays

CSR assays were performed as described previously (31). Briefly, cells were seeded in the presence of 1 μg/ml anti-CD40 antibody (16-0402-86; bioscience), 5 ng/ml of IL-4 (404-ML; R&D Systems), and 0.5 ng/ml TGF-β1 (R&D Systems 240-B) and grown for 72 h. Cells were stained with a FITC-conjugated anti-mouse IgM antibody (BD Biosciences 553437) and anti-mouse IgA (BD biosciences 556969) conjugated with CF633 (Biotium ‘Mix-n-Stain’ kit) for 90 minutes at 4°C. Stained cells were analyzed on a LSR II flow cytometer (BD Biosciences). CSR efficiency is determined as the percentage of IgA-positive cells.

### Amplicon sequencing and microhomology analysis of CRISPR/Cas9-induced chromosomal DSBs

We have previously used a CRISPR/Cas9 strategy to sequence DSBs from within the FancG locus (32). To induce deletional DSBs, two gRNA/Cas9/puro plasmids were transfected into 293T cells with the indicated genotypes. After 48 hours, cells were placed on selection with complete media + 2 μg/ml puromycin. Cells were harvested and DNA prepared 72 h later and PCR performed to detect chromosomal deletions. PCR fragments were separated by size on an agarose gel and purified using a QIAquick gel extraction kit (Qiagen). Amplicon sequencing was performed by Genewiz on an Illumina MiSeq (running in 2 × 250 paired end mode). Analysis to classify microhomology usage and indel size was performed using a Python script generously provided by Dale Ramsden and Adam Luthman (UNC Chapel Hill) (33).

### Mammalian expression vectors and recombinant protein expression constructs and purification

XLF was produced in bacteria and Ku in insect cells as described (32). The Lig4/XRCC4 complexes (WT, K273A, and 5XK) were produced in bacteria as described (12). All proteins batches were dialyzed against 150 mM KCl, 20 HEPES pH 8, 1 mM EDTA, 2 mM DTT and 10% (v/v) glycerol, flash frozen in liquid nitrogen and stored at −80°C. T4 DNA ligase was obtained from New England Biolabs. Mammalian expression constructs for wild-type Lig4 and Lig4-K273A were generated by subcloning from plasmids described above. The Lig4 5XK mutant was prepared by subcloning a geneblock (IDT) encoding K273A, K449R, K451R, K352R and K345R via PflMI/BlpI subcloning. Detailed purification procedures for Lig1 and nuclear Lig3/XRCC1 will be reported elsewhere. Briefly, Lig1 and Lig3/XRCC1 were produced as MBP N-terminal fusions after overexpression in suspension 293T cells and purified over amylose resin followed by chromatography through S Sepharose and stored at –80°C in 250 mM KCl, 20 mM Tris pH 7.5, 1 mM EDTA, 1 mM DTT and 10% (v/v) glycerol.

### Ligation assay

Ligation assays were performed in a volume of 10 μl containing 50 ng of linearized pUC19 plasmid DNA (with XbaI for cohesive ends and SmaI for blunt ends), 1 mM ATP, 1 mM DTT, 20 mM Tris–HCl pH8.0, 2 mM MgCl_2_, and 60 mM KCl, 1% (v/v) glycerol with addition of proteins at the indicated concentrations. Reactions were incubated at room temperature for 30 min before addition of Proteinase K at 1.4 mg/ml final concentration and 1× of a loading buffer containing 0.01% SDS (NEB #B7024S) and incubated for 10 min at 55°C. Samples were next resolved by 0.8% agarose gel electrophoresis in Tris-Borate-EDTA buffer. Gels were stained in Tris-Borate-EDTA buffer supplemented with 0.5 μg/ml ethidium bromide and destained in deionized water. Images were captured under UV light using a Bio-Rad chemidoc and quantitatively analyzed with ImageJ.

### Oligonucleotides and antibodies

Oligonucleotides used in this study are as follows; only one strand of the oligonucleotides used for targeting PAM sites are presented and are without BbsI overhangs.

For 293T Lig4 CRISPR experiments:

gRNA Lig4: CATACGTTCACCATCTAGCT

Lig4 K273A HDR:

CTATTGCAGATATTGAGCACATTGAGAAGGATATGAAACATCAGAGTTTCTACATAGAAACAGCGCTAGATGGTGAACGTATGCAAATGCACAAAGATGGAGATGTATATAAATACTTCTCTCGAAATGG

For U2OS CRISPR experiments:

gRNA Lig4: GTTCAGCACTTGAGCAAAAG.

The U20S clone used in the experiments had + 1 frameshift mutations on both alleles.

For 293T FANCG CRISPR experiments:

gRNA1 FancG:GGGCCAGGCCTGGGTTCAAC

gRNA2 FancG:GACTTAAGAGAAAGGGACTG

5′ FancG PCR: CCCAAGATGTCCCGGCTGTGGG

3′ FancG PCR:CCATGGGCCTCTCTGTCCTTGCAC

Oligonucleotides for substrate PCR:

5′ coding joint PCR: CGGTGGGAGGTCTATATAAGCA

3′ coding joint PCR:CTACACCGTGGTGGAGCAGTA

5′ signal joint PCR:ACCTTGAAGCGCATGAAGGGC

3′ signal joint PCR:TCCATGCGGTACTTCATGGTC

Antibodies utilized in this study include: anti-DNA Ligase IV (Abcam, 193353), anti-Ku80 (Invitrogen, 111), anti-DNA-PKcs (generous gift Tim Carter).

## RESULTS

### Cells expressing catalytically inactive Lig4 or completely deficient in Lig4 are markedly sensitive to DSB-inducing agents

**Figure 1.**
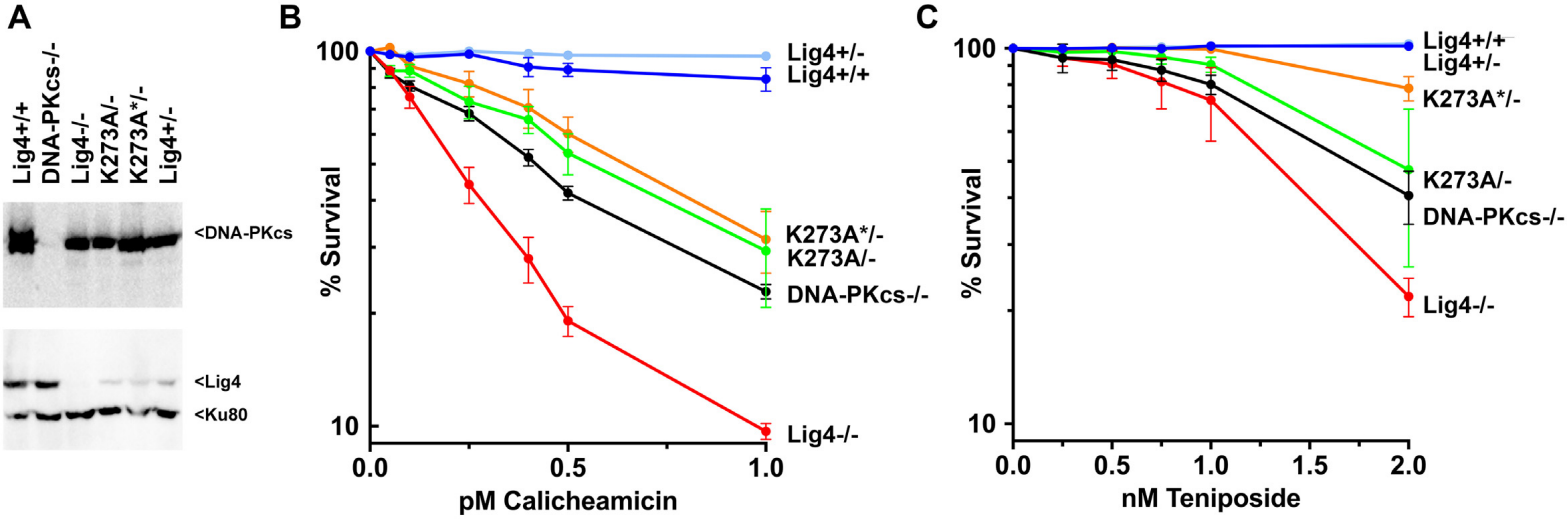
Cells expressing catalytically inactive Lig4 or completely deficient in Lig4 are markedly sensitive to the DSB-inducing agents calicheamicin and teniposide. **(A)** Western blot of Lig4 in wild-type 293T cells (Lig4+/+), heterozygous cells (Lig4+/−), or 293T cells deficient in Lig4 (Lig4−/−), deficient in DNA-PKcs (DNA-PKcs−/−) or with mutations in Lig4 [K273A/−, and K273A*/−]. (These are two independent clones possessing one copy of catalytically inactive Lig4 and a frameshift mutation on the second Lig4 allele; the K273A clone containing an additional mutation, I270V is labelled K273A*/−, for brevity.). **(B)** Sensitivity of the same panel of cell strains to calicheamicin **(B)** or teniposide **(C)** was assessed. Briefly, cells were plated in 24 well plates into complete medium with increasing doses of calicheamicin or teniposide. After 7 days, MTT was added, and cell survival assessed by colorimetry. Survival assays were performed four times in duplicate (B) or three times in duplicate (C).

To begin to address whether Lig4 has a non-catalytic function in living cells, a CRISPR/Cas9 strategy was devised using a single gRNA that targets the codon AAG, encoding K273 [the well-conserved catalytic site (15,34,35)] in Lig4 and a single stranded oligonucleotide to direct an HDR mediated K273A mutation at this site (as well as the introduction of a novel HhaI site). From these transfections in 293T cells, single clones were isolated, and DNA was isolated for PCR/HhaI digestion. Amplicon sequencing was performed on clones that included addition of the HhaI site in the initial PCR screen. Four clones were chosen for further analyses (Supplementary Table S1). Sanger sequencing from one clone (clone 17, that lacks a wild-type allele by initial PCR screen) revealed two alleles with frameshift mutations, and we conclude that this clone is completely deficient in Lig4. Amplicon sequencing of two clones (that have a novel HhaI site ascertained by the initial PCR screen) reveal that one (clone 144) has the targeted K273A mutation on one allele, and a frameshift mutation on the second allele. The second clone (clone 195) contains the K273A mutation as well as an additional mutation, I270V on one allele, and the second allele includes a frameshift mutation (Supplementary Table S1). A heterozygous clone (clone 104) was isolated that has an inactivating frameshift mutation on one allele, but the second allele is wild-type (Figure 1A).

Cells with defects in the NHEJ pathway are markedly sensitive to numerous different DNA damaging agents that can induce DSBs by various mechanisms. We chose two drugs, calicheamicin [generating DSBs with 3′ overhangs with 3′ phosphoglycolate (3′PG)] (36) that generates DSBs throughout the cell cycle (36) and teniposide a topoisomerase II (topII) inhibitor that generates two-ended DSBs primarily in S phase [with 5′ overhangs] (37). Cellular calicheamicin and teniposide sensitivity was assessed for wild-type 293T as well as Lig4+/−, K273A/−, K273A + I270V/− (labelled K273A*/−, for brevity), and Lig4−/−, clones as well as a previously described DNA-PKcs deficient clone (38) using MTT staining as a measure of cellular viability (Figure 1B, C). As can be seen, cells completely deficient in Lig4 are remarkably sensitive to calicheamicin and teniposide; the two clones expressing only catalytically inactive Lig4 are also sensitive to both drugs, but substantially more resistant than cells that are completely deficient in Lig4. In contrast, at these doses, the Lig4+/− heterozygous clone is similarly resistant to both drugs as wild-type 293T cells. Finally, the previously described DNA-PKcs−/− 293T cells are also hypersensitive to calicheamicin and teniposide, but more resistant than Lig4−/− cells consistent with previous studies (22). These data suggest that catalytically inactive Lig4 promotes survival (albeit inefficient) to calicheamicin and teniposide.

### Catalytically inactive Lig4 promotes joining of DSBs on episomal substrates in 293T cells

**Figure 2.**
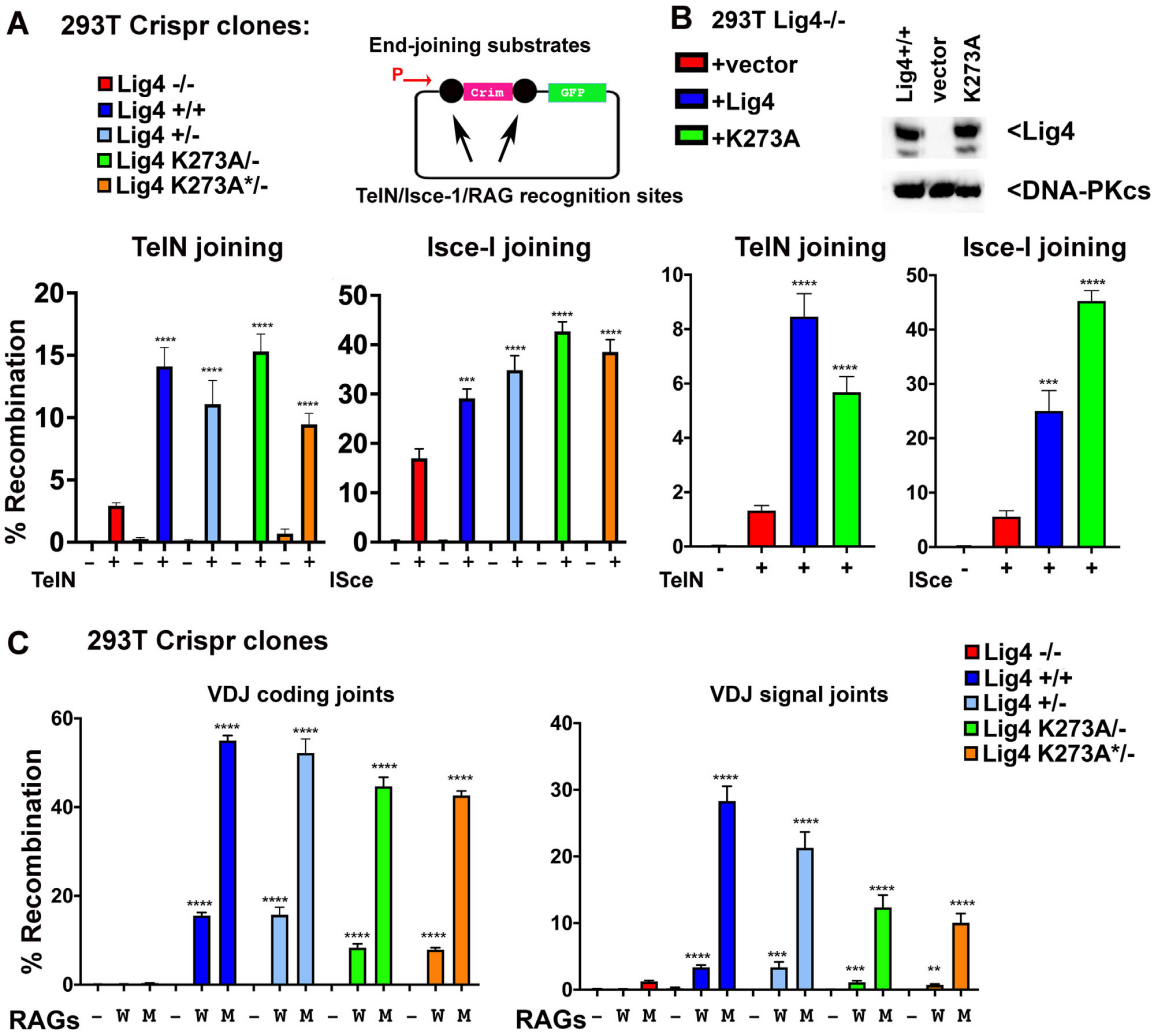
Catalytically inactive Lig4 promotes joining of DSBs in episomal substrates in 293T cells. **(A)** Fluorescent substrates (depicted in top panels indicate promoter with arrow) were utilized to detect TelN, I-SceI, or VDJ coding and signal joints in 293T cells of the indicated genotypes. 293T clones with the indicated genotypes were co-transfected with TelN or I-SceI plasmid substrate, with and without enzyme (−/+) and analyzed for red/green fluorescence via flow cytometry (lower panels). (**B)** Lig4−/− 293T cells were tested for TelN and I-SceI joining by co-transfecting the TelN/I-SceI substrates and expression plasmids with or without co-transfection of expression plasmids encoding either WT or catalytically inactive human Lig4 as indicated. (**C)** 293T clones were co-transfected with wild-type (W) or hypermutant (M) RAG expression plasmids with either plasmid substrates to detect coding or signal end-joining as indicated by analysis of red/blue fluorescence via flow cytometry. Episomal VDJ assays testing joining of coding (hairpin) and signal (blunt) ends. Cells were transfected with substrate and either: no Rag2 (−), WT rags (W), or mutant rag2 (M) and analyzed via flow cytometry. In A, B, and C, student's T test comparing joining rates between Lig4−/− and either Lig4+/+, Lig4+/−, or K273A were performed; ****P < 0.0001; ***P < 0.001; ns = not significant in two-tailed unpaired *t* test.

We have previously developed plasmid substrates with recognition sequences for DNA endonucleases that can produce a range of DNA end structures. The cutting sites flank the coding sequence for a red fluorescent protein (Crimson, RFP) driven by a CMV promoter and by co-transfecting substrate with appropriate enzymes, end-joining efficiency can be assessed by measuring the fraction of cells that express GFP or CFP after excision of the RFP cassette. The TelN protelomerase and I-SceI homing endonuclease produce hairpin DNA ends or 4 nucleotide overhangs respectively. To test whether 293T cells that either lack Lig4 entirely or retain a single copy of a catalytically inactive Lig4 gene can rejoin TelN or I-SceI-induced DSBs, a series of episomal end-joining assays were performed (Figure 2). Our previous work has shown that cells with defects in either the DNA-PK complex, Artemis, or the Lig4 complex are all significantly and similarly impaired in joining TelN-induced hairpin ends, even though hairpin ends are opened in cells deficient in the Lig4 complex (that retain DNA-PK and Artemis), but are obviously sealed in cells lacking DNA-PK or Artemis(20). Consistent with this previous study, cells deficient in Lig4 have a marked decrease in TelN joining (Figure 2A). In contrast, rejoining I-SceI DSBs is only ∼50% reduced in cells deficient in Lig4, because restriction enzyme-induced DSBs are available to the a-EJ pathway [consistent with previous studies (25,26,39)]. These data suggest that engagement of the Artemis/DNA-PK complex to resolve the closed hairpins must in some undefined way restrict the opened hairpins to the NHEJ pathway. Strikingly, in contrast to Lig4 deficient cells, the two independent clones expressing only catalytically inactive Lig4 rejoin significant levels of both I-SceI and TelN-induced DSBs (Figure 2A). These data suggest that the Lig4 complex (whether active or not) promotes end-joining by a-EJ presumably by relaxing the NHEJ restriction imposed by hairpin opening by DNA-PK/Artemis.

To confirm that the observed differences in end-joining are the result of the targeted Lig4 mutations, we utilized the Lig4 deficient 293T cells and expression vectors encoding wild-type or catalytically inactive human Lig4 (Figure 2B) in a complementation experiment. As can be seen, both wild-type and catalytically inactive Lig4 (but not empty vector) substantially reverse both TelN and I-SceI joining in 293T cells completely deficient in Lig4. In cells that lack NHEJ, DSBs—including those induced by restriction enzymes, introduced during class switch recombination, or by CRISPR/Cas9—can all be efficiently re-joined by the a-EJ pathway. In contrast, NHEJ-deficient cells do not join VDJ-associated DSBs because VDJ recombination intermediates are tightly restricted to the NHEJ pathway by mechanism(s) that are still incompletely understood. To corroborate the TelN joining experiments, we next assessed VDJ coding and signal end-joining in the same panel of 293T cells. In these experiments we used both wild-type RAG expression vectors as well as a well-studied hyper-RAG2 mutant that substantially increases VDJ joining by de-stabilizing the RAG post-cleavage complex(s) that function to limit end-joining to the NHEJ pathway (40). As can be seen, 293T cells lacking Lig4 are effectively incapable of joining RAG-induced DSBs, either blunt-ended signal ends or hairpin coding ends. In contrast, cells expressing catalytically inactive Lig4 join both coding and signal ends at levels only modestly reduced as compared to levels observed in wild-type 293T or Lig4+/− 293T cells. The hyper RAG2 mutant substantially increases the level of both coding and signal joining in wild-type cells, but joining is minimal in cells completely deficient in Lig4. In contrast, both signal and coding end-joining is robust, and only modestly reduced in cells expressing catalytically inactive Lig4 in experiments utilizing the hyper-RAG mutant (Figure 2C). It is interesting to note that cells expressing catalytically inactive Lig4 have a more severe deficit in signal end-joining than in coding end-joining. To our knowledge, this is unlike VDJ deficits in any other NHEJ mutant studied to date, and perhaps suggest that catalytically inactive Lig4 can promote joining of over-hanged DNA ends better than blunt DNA ends. In sum, we conclude that catalytically inactive Lig4 efficiently promotes rejoining of RAG-induced DSBs. These data reveal that once VDJ intermediates are released to a complete Lig4 complex (potentially, the short-range NHEJ complex), the strict requirement for NHEJ-only dependent joining of RAG-induced DSBs has been fulfilled, and the RAG-induced DSBs can be joined by the a-EJ ligases just as well as non-RAG induced DSBs.

### Catalytically inactive Lig4 promotes joining of DSBs on episomal substrates in diverse cell types

**Figure 3.**
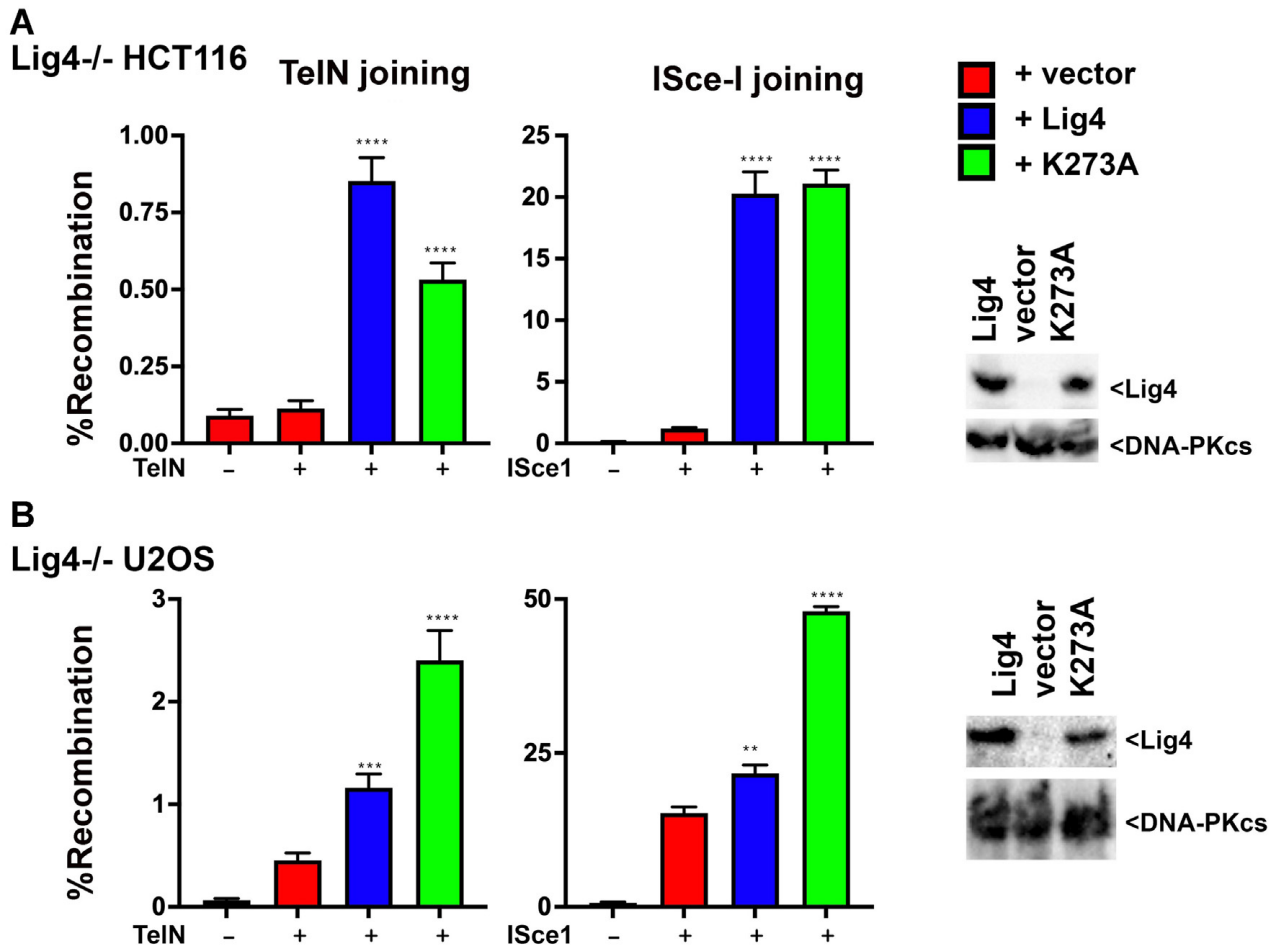
Catalytically inactive Lig4 promotes joining of DSBs in episomal substrates in a variety of cell types. (**A)** Lig4−/− HCT116 cells were tested for TelN and I-SceI joining by co-transfecting the TelN/I-SceI substrates and expression plasmids with or without co-transfection of expression plasmids encoding either WT or catalytically inactive human ligase IV as indicated. (right) Immunoblot of cells transfected with indicated expression plasmids, and probed for either Lig4 or DNA-PKcs. (**B)** Lig4−/− U20S cells were tested for TelN and I-SceI joining by co-transfecting the TelN/I-SceI substrates and expression plasmids with or without co-transfection of expression plasmids encoding either WT or catalytically inactive mouse Lig4 as indicated. (right) Immunoblot of cells transfected with indicated expression plasmids, and probed for either Lig4 or DNA-PKcs. In A and B, student's T test comparing joining rates between vector and Lig4 or K273A were performed; ****P < 0.0001; ***P < 0.001; **P < 0.01; ns = not significant in two-tailed unpaired *t* test.

To extend these studies, a CRISPR/Cas9 strategy was utilized to ablate Lig4 from the U2OS cell strain, and a Lig4 deficient HCT116 cell strain was obtained from Dr. Eric Hendrickson (41). As can be seen, in these cell types, both wild-type and catalytically inactive Lig4 (but not empty vector) substantially reverse both TelN and I-SceI end-joining (Figure 3), completely analogous to the results observed in Lig4−/− 293T cells. These data substantiate our conclusion that the Lig4 complex facilitates end-joining of a variety of DSBs on episomal substrates.

### Catalytically inactive Lig4 supports chromosomal rearrangement during class switch recombination

Previously we studied the role of Lig4 in the process of immunoglobulin class switch recombination (CSR) using a well-studied mouse B cell model, CH12F3 cells (31). Retroviral vectors encoding either wild-type or K273S murine Lig4 were prepared and used to transduce a previously described Lig4−/− CH12F3 cell strain. When CH12F3 cells are stimulated with an anti-CD40 antibody, interleukin 4, and TGFβ1 (+CIT), CSR (from IgM to IgA) is robustly induced. This produces clear populations of IgM+/IgA-, IgM+/IgA+ and IgM−/IgA + cells that can be analyzed by flow cytometry. Like VDJ recombination, CSR is a lymphocyte specific DNA recombination event that proceeds through a double-strand break intermediate; but unlike VDJ recombination, a-EJ can facilitate CSR (albeit at reduced levels) in the absence of most NHEJ factors. Our previous study demonstrated that either Lig1 or Lig3 could facilitate a-EJ in a Lig4 deficient murine B cell line (CH12F3 cells) that were induced to undergo class switch recombination.

**Figure 4.**
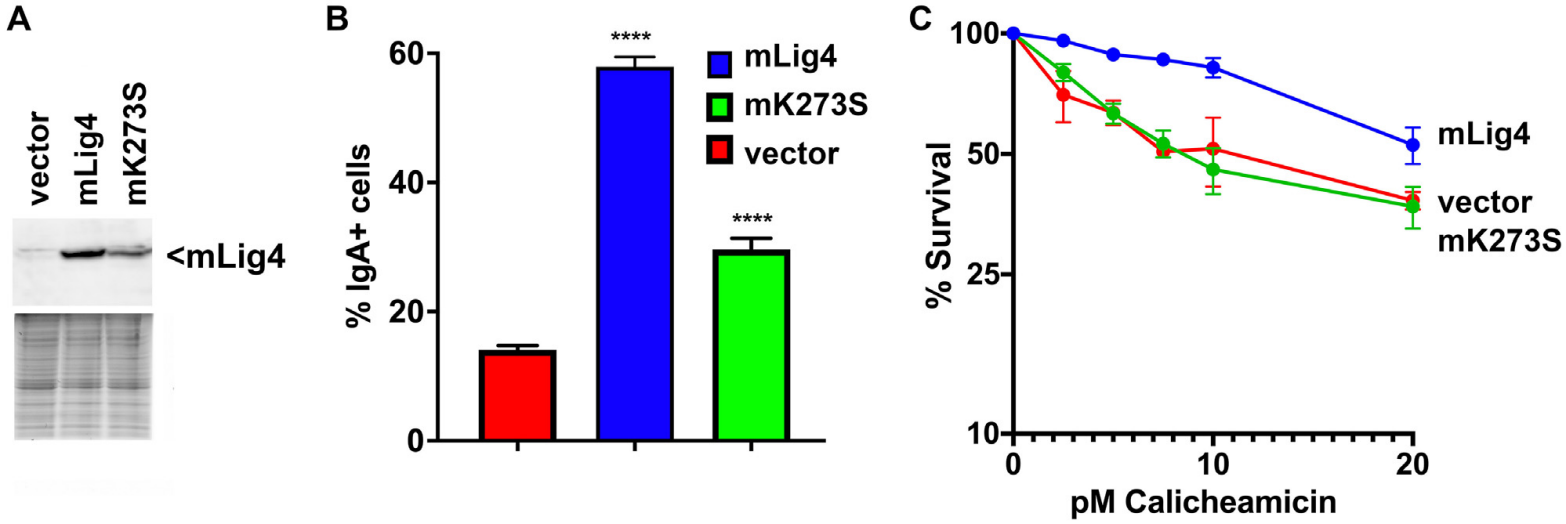
Catalytically inactive Lig4 is only moderately deficient in CSR. **(A)** Western blot of murine Lig4 expressed in Lig4−/− CH12F3 cells. **(B)** CSR efficiency (percentage of IgA-positive cells, assessed by flow cytometry) in mouse CH12F3 cells expressing WT Lig4, catalytically inactive Lig4 (dLig4), or no Lig4. Error bars indicate SE of three independent experiments. **(C)** Sensitivity of the same panel of cell strains to calicheamicin was assessed. Briefly, cells were plated in 24 well plates into complete medium with increasing doses of calicheamicin. After 7 days, MTT was added, and cell survival assessed by colorimetry. In B, student's T test comparing joining rates between vector and Lig4, or vector K273S were performed; ****P < 0.0001; in two-tailed unpaired t test. Survival assay was performed three times in duplicate.

We next assessed CSR in the CH12F3 Lig4−/− cells expressing wild-type Lig4, K273S, or vector only from lentivirus expression vectors (Figure 4A). Consistent with our previous study, only ∼10% of Lig4 deficient CH12F3 cells expressing vector alone undergo CSR compared to ∼60% of cells expressing wild-type murine Lig4. Cells expressing K273S Lig4 display an intermediate level of CSR (Figure 4B). We conclude that catalytically inactive Lig4 promotes chromosomal end-joining of cell-programmed DSBs. We also assessed cellular resistance to calicheamicin in this panel of CH12F3 cells. As can be seen, whereas cells expressing wild-type Lig4 are substantially more resistant to calicheamicin than cells expressing no Lig4, cells expressing catalytically inactive Lig4 are similarly sensitive to calicheamicin as are cells lacking Lig4 (Figure 4C). We conclude that catalytically inactive Lig4 cannot restore cellular resistance to DNA damaging agents in all cell types.

### Catalytically inactive Lig4/XRCC4/XLF promotes Lig3-mediated joining of both cohesive and blunt DNA ends *in vitro*

**Figure 5.**
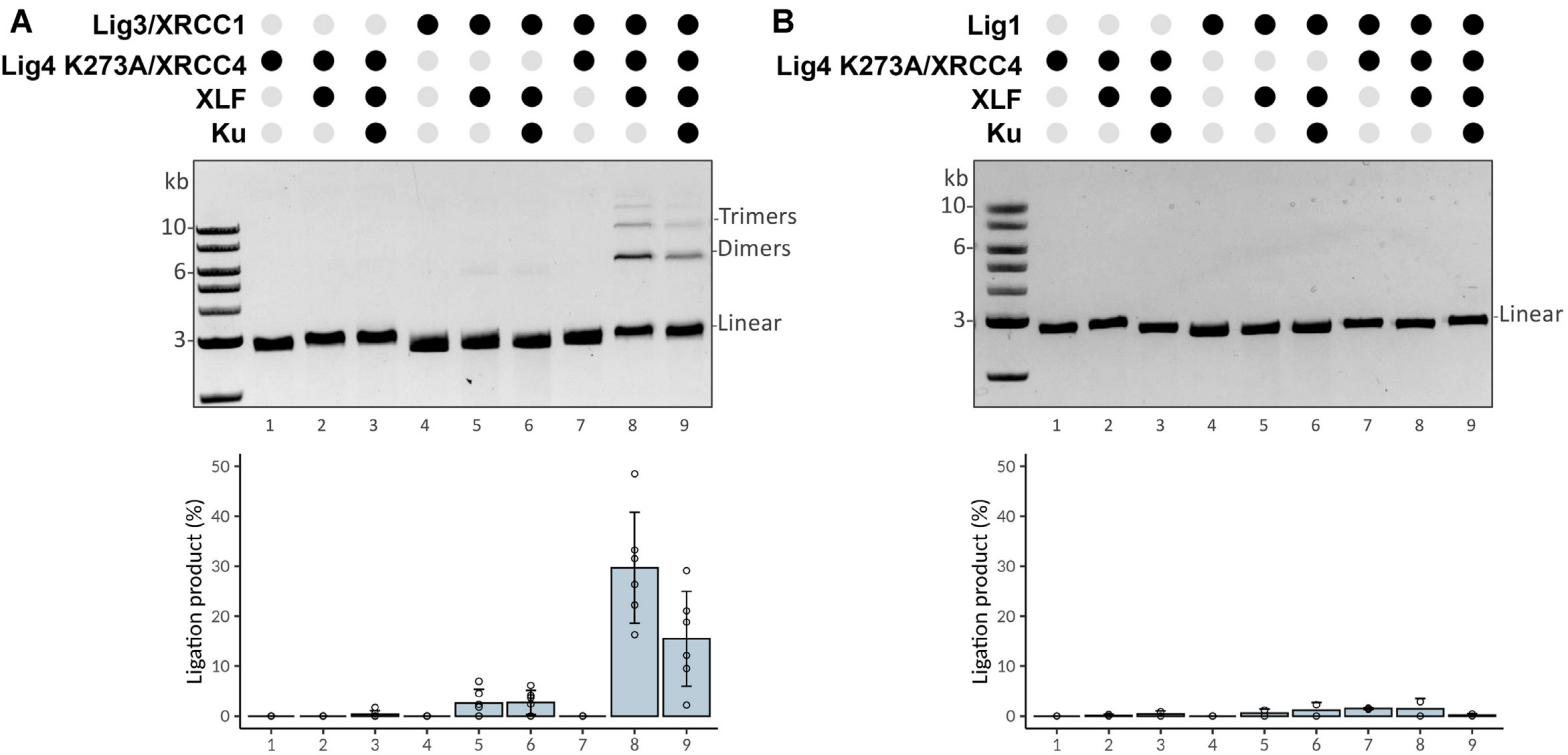
Catalytically inactive Lig4/XRCC4/XLF promotes Lig3-mediated joining of blunt DNA ends *in vitro*. The capacity for catalytically inactive Lig4/XRCC4 to promote ligation by Lig3 **(A)**, or Lig1 **(B)** was assessed by incubating a blunt DNA ligation substrate (linearized pUC19, 2.7 kb) with purified recombinant proteins as indicated at the following concentrations: Ku, 250 nM; XLF, 200 nM; Lig4/XRCC4, 500 nM; Lig3/XRCC1, 40 nM; and Lig1,180 nM. Reactions (10μl) containing 50 ng of linear DNA were incubated for 30 minutes at room temperature, deproteinized, analyzed by agarose gel electrophoresis. Bar graph represents six independent experiments and error bars represent SD.

The robust joining facilitated by catalytically inactive Lig4 in cellular assays suggests that the NHEJ short-range complex might be capable of facilitating ligation of DNA ends by either Lig3 or Lig1. To directly test this possibility, Lig1 and Lig3/XRCC1 were over-expressed and purified from human 293T cells (Supplementary Figure S1A) and tested for activity (Supplementary Figure S1B). Then purified Lig1 or Lig3/XRCC1 were tested in ligase assays that included the components of the NHEJ short range complex (Ku, Lig4/XRCC4, and XLF) using catalytically inactive Lig4, on substrates with either blunt or cohesive DNA ends. In these assays, higher levels of Lig4/XRCC4 were used as compared to either Lig1 (2.8 molar excess) or Lig3/XRCC1 (12.5 molar excess). As can be seen (Figure 5A), using a blunt ligation substrate, no ligation products are observed with just the NHEJ components, and no activity is observed with only Lig3/XRCC1. In contrast, when Lig3/XRCC1 is added to reactions containing catalytically inactive Lig4 (with or without Ku), robust ligation is observed. This activity is completely dependent on both Lig4/XRCC4 and XLF, but Ku is dispensable. In parallel assays, Lig1 activity is marginally enhanced by catalytically inactive Lig4 complex (Figure 5B) under any of the conditions tested.

**Figure 6.**
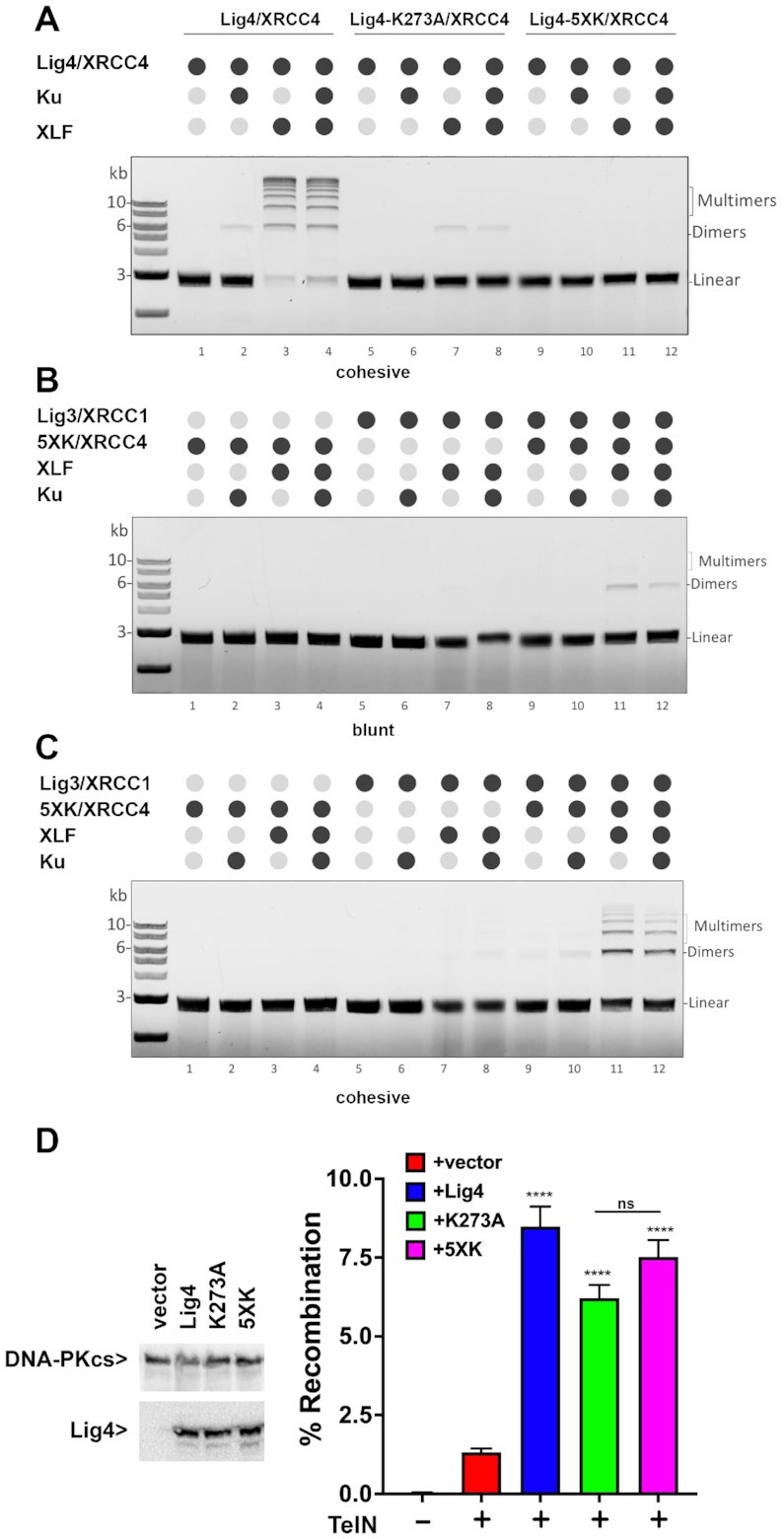
The 5XK Lig4 mutant has no residual ligation activity but stimulates Lig3 joining in vitro, and promotes end-joining in living cells. **(A)** DNA ligation assay using a linear DNA fragment (2.7 kb) with cohesive ends and NHEJ components as indicated. **(B + C)** Lig3 stimulation assay with 5XK mutant using a linear DNA fragment (2.7 kb) with **(B)** blunt ends or **(C)** cohesive ends. Assays were performed using 250 nM 5XK Lig4 and Ku, 200 nM XLF and 40 nM Lig3 as final concentration. Ligation products were deproteinized and resolved by agarose gel electrophoresis before staining with ethidium bromide and detection. **(D)** (left) Immunoblot of cells transfected with indicated expression plasmids, and probed for either Lig4 or DNA-PKcs. (right) Lig4−/− 293T cells were tested for TelN joining by co-transfecting the TelN substrates and expression plasmids with or without co-transfection of expression plasmids encoding either WT, K273A, or 5XK mutant. Student's T test comparing joining rates between vector and Lig4, K273A or 5XK were performed; ****P < 0.0001; ***P < 0.001; **P < 0.01; ns = not significant in two-tailed unpaired *t* test.

This assay was repeated using DNA with cohesive ends (Supplementary Figure S2); as can be seen, ligation of compatible ends is robust in the presence of catalytically inactive Lig4 complex and Lig3/XRCC1, but not Lig1, suggesting that catalytically inactive Lig4 complex can stimulate Lig3/XRCC1-mediated joining of cohesive ends. However, (to our surprise) we consistently observed very low levels of ligation products in reactions with XLF and XRCC4/Lig4 (catalytically inactive, K273A) without Lig3/XRCC1 or Lig1. The activity represents <5% the activity observed in assays with wild-type Lig4 complexes (compare Supplementary Figure S3 bottom panel and Supplementary Figure S2A, B lanes 2 and 3; also see Figure 6A). This residual activity could not be attributed to a contaminating ligase since the proteins are prepared in *E. coli* that has only NAD dependent ligase and was not due to contaminant ligase activity in the XLF and Ku protein preparations (Supplementary Figure S3). Moreover, adenylation assays demonstrate minimal adenylation of the K273A mutant Lig4 (Supplementary Figure S4). From the recent cryo-EM study of the short-range complex (4) that docked a high resolution structure of Lig4 into the complex (42), we observed that 4 additional lysine residues are very close to the 5′ phosphate of the DNA end; whereas K273 is ∼7 Å away, lysines 449, 451, 352 and 345 are between 8 Å and 9.5 Å away from the 5′ phosphate. We considered that one of these four lysines might serve as a back-up adenylation site explaining the minimal ligase activity of the K273A mutant. These four residues were substituted with arginine in the K273A mutant construct. As can be seen, this 5× lysine mutant (Lig4-5XK) has no residual activity in ligase assays (Figure 6A) but retains the capacity to stimulate Lig3 activity towards both blunt and cohesive ends *in vitro* (Figure 6B, C) to a similar extent as the K273A mutant. Moreover, in cellular assays, the 5× lysine mutant joining activity that is indistinguishable from that of the K273A mutant (Figure 6D). From these data, we conclude that the catalytically inactive Lig4/XRCC4/XLF complex promotes end-joining by Lig3 *in vitro*, potentially explaining the robust joining activity observed in living cells expressing the catalytically inactive complex.

### Cells expressing K273A Lig4 cannot perform fill-in end-processing, but robustly promote a-EJ in episomal assays

Clearly catalytically inactive Lig4 enhances end-joining of episomal substrates and in some cell types, cellular survival after exposure to agents that induce DSBs. To gain knowledge as to how end-joining is enhanced by the catalytically inactive Lig4 complex, we used assays that clarify structural characteristics of repaired DSBs. More specifically, we focused on determining whether catalytically inactive Lig4 promotes end-joining that is similar to authentic NHEJ [i.e. generating joints ranging from perfect end-joining with no or minimal base pair loss, or joining at sites of MH (1–3bp)], or alternatively, if catalytically inactive Lig4 promotes joining that is more similar to a-EJ [characterized by increased loss of terminal nucleotides, and a strong dependence on the presence of MH (often longer than 3bp)].

**Figure 7.**
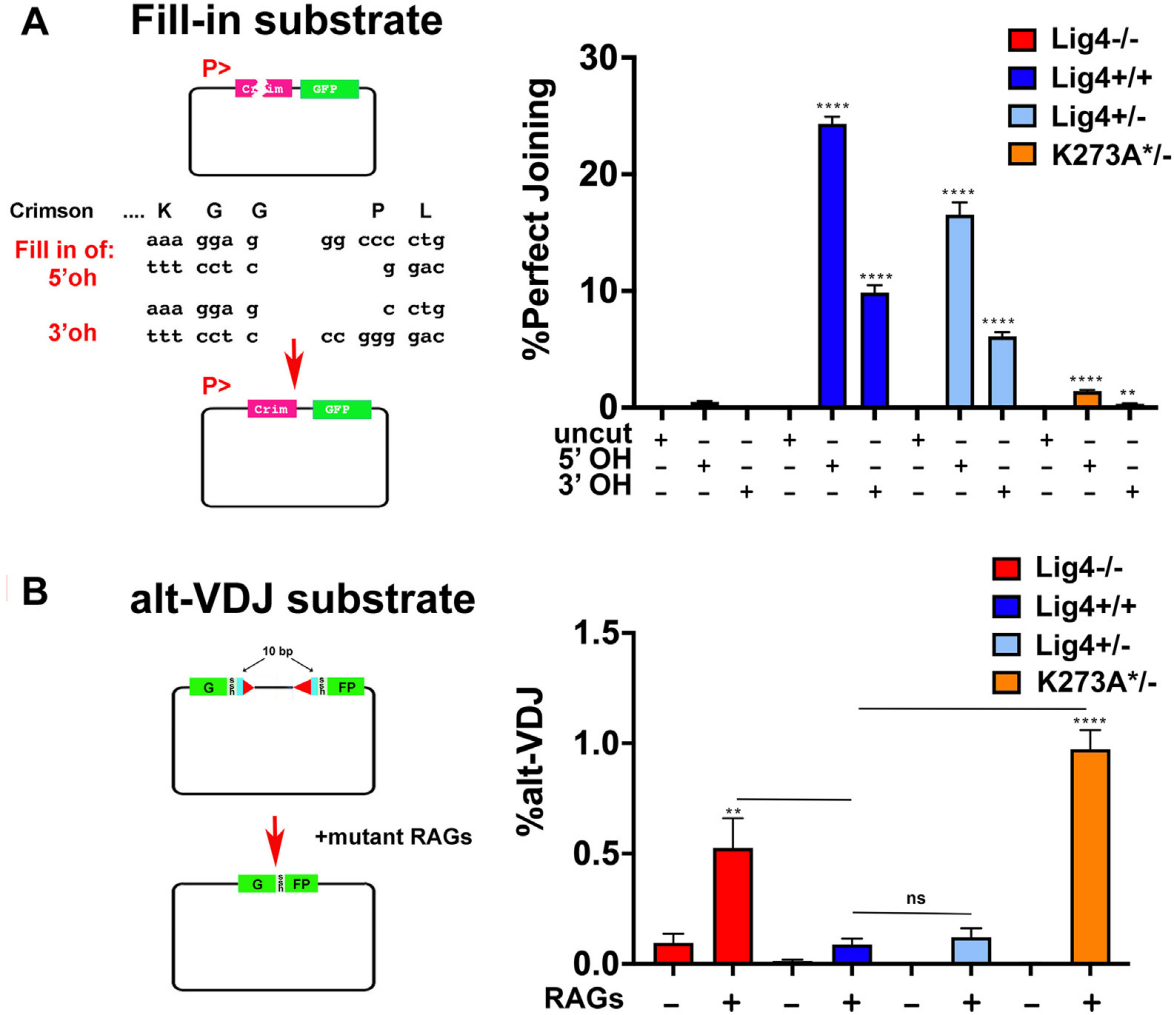
DSBs repaired in cells expressing K273A Lig4 have characteristics of a-EJ. **(A)** 293T clones of the indicated genotypes were transfected with Fill-in joining substrate either uncleaved or cleaved with Eco53KI and PspOMI (to generate blunt and 5′ overhangs), or Eco53KI and ApaI(to generate blunt and 3′ overhangs). Cells were assessed for RFP and GFP expression 72 hours later. Student's T test comparing joining rates between Lig4−/− and either Lig4+/+, Lig4+/−, or K273A*/− were performed; ****P < 0.0001; in two-tailed unpaired t test. **(B)** 293T clones of the indicated genotypes were co-transfected with the alt-VDJ substrate, a ds-RED expression plasmid, and hypermutant RAG expression plasmids. Restoration of the GFP reading frame requires joining via a 9bp region of MH that occurs 10bp from the termini of both coding ends. Cells were assessed for RFP and GFP expression 72 hours after transfection. Student's T test comparing joining rates between Lig4−/− and either Lig4+/+, Lig4+/−, or K273A*/− as well as between Lig4+/+ and either Lig4+/− or K273A*/− were performed; ****P < 0.0001; ***P < 0.001; **P < 0.01; in two-tailed unpaired *t* test.

Although NHEJ is generally characterized as an error-prone repair mechanism, numerous studies have documented the fidelity of NHEJ when joining most DSBs (9,10). For example, perfect joining of compatible ends are highly favored and incompatible ends are rejoined to minimize nucleotide loss (9,10). To assess the accuracy of end joining mediated by catalytically inactive Lig4 we designed an episomal substrate that measures fidelity of repair. Briefly, the substrate plasmid is restricted with two enzymes to generate a blunt end on one side and an over-hanged end (either 5′ or 3′) on the other side as illustrated (Figure 7A). To restore the Crimson open reading frame, the ends must be aligned so that the missing bases across from the overhangs are filled in. In both cellular and *in vitro* models of NHEJ, perfect fill-in of DNA ends like these is efficiently mediated by NHEJ (9,10). If uncut plasmid is transfected, Crimson is not expressed because of the disruption in the open reading frame, but GFP is expressed by use of its own ATG. When restricted plasmids are transfected, if any plasmid rejoining occurs, GFP is expressed. With this assay, ‘Perfect rejoining’ represents the percent GFP positive cells that also express Crimson, and in these assays, GFP expression is robust in all cell types. As would be expected, cells completely lacking Lig4−/− are completely unable to perfectly rejoin the transfected substrate promoting Crimson expression, whereas in wild-type 293T cells >30% of the cells that rejoin the plasmid, rejoin the plasmid perfectly and Crimson is robustly expressed (Figure 7A). In contrast to other joining assays (Figure 2), cells expressing only catalytically inactive Lig4 are similarly deficient in perfect rejoining as are cells completely lacking Lig4. We conclude that catalytically inactive Lig4 cannot promote perfect rejoining of the fill-in episomal substrate. It follows that the catalytically inactive complex either does not efficiently support fill-in end-processing, or that the cellular ligase facilitating end-joining (Lig3 or Lig1) does not efficiently join the filled-in ends.

To assess whether a-EJ-like events facilitate repair in cells expressing catalytically inactive Lig4, we utilized an assay developed by Roth *et al.* (30). It is well-appreciated that short sequence homologies at opened coding-end termini can facilitate coding end-joining (43); these short sequence homologies are generally small (1–3 base pair) and occur close to the DNA terminus. We utilized their a-EJ assay (Figure 7B), termed alt-VDJ, using mutant RAG expression constructs that destabilize the RAG post-cleavage complex(s) to promote more alt-VDJ) to determine whether cells expressing catalytically inactive Lig4 depend on MH for joining. This assay requires nucleotide deletions of 10bp from each coding end, and then use of 9 base pair of short sequence homology to restore the GFP open-reading frame. Whereas minimal alt-VDJ is observed in wild-type 293T cells or Lig4+/− heterozygous cells where end-processing generally precludes loss of the required 10bp from each coding-end, alt-VDJ is readily detected in Lig4−/− 293T cells (Figure 7B). Strikingly, alt-VDJ joining is robust in cells expressing catalytically inactive Lig4; these data suggest that K273A mutant Lig4 promotes end-joining that is similar to a-EJ.

### Rejoined episomal DSBs in cells expressing K273A Lig4 have characteristics of a-EJ mediated joining

**Figure 8.**
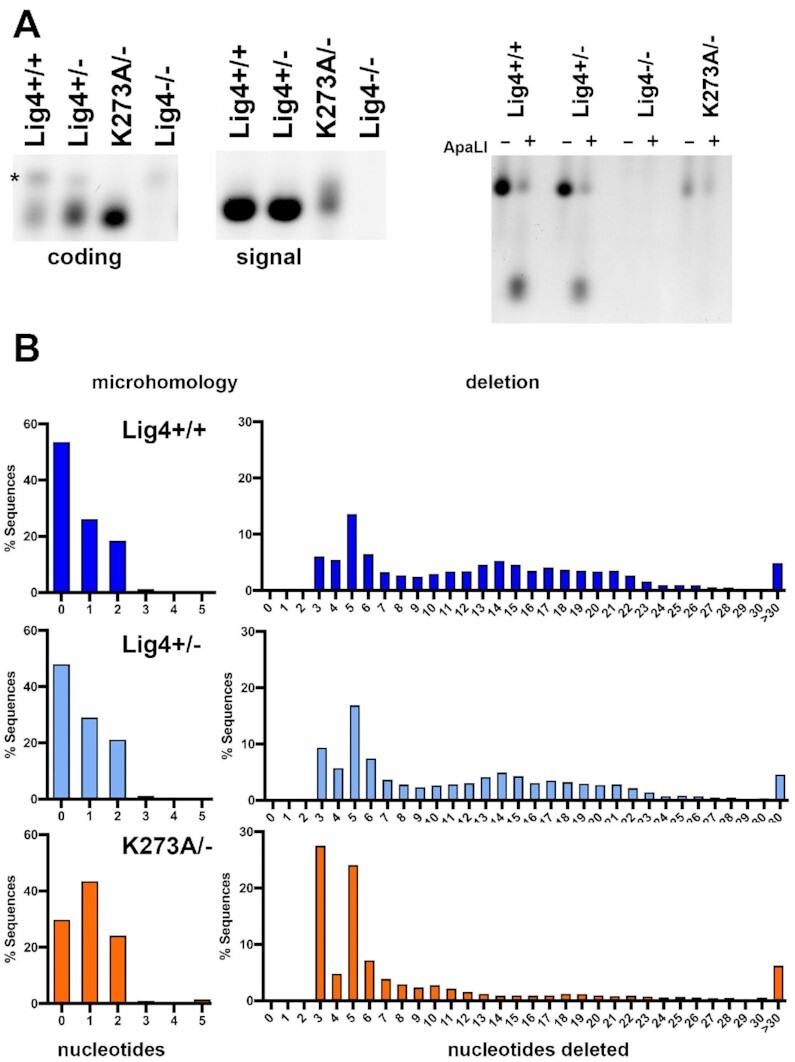
Rejoined episomal VDJ coding and signal joints in cells expressing K273A Lig4 have characteristics of a-EJ mediated joining. **(A)** Coding (left) and signal joints (middle) were PCR amplified from substrates isolated from cells of the indicated genotypes 72 h after transfection. (*) indicates a PCR artifact. Half of the PCR reaction for signal joint amplification was digested with ApaLI prior to electrophoresis (right). **(B)** Isolated coding joints from the indicated cell strains were subjected to amplicon sequencing. Histograms depicting numbers of nucleotides deleted or extent of microhomology at site of joining.

To further characterize DSB joining events in cells expressing catalytically inactive Lig4, we characterized VDJ joints isolated from cells expressing the inactive complex. In cells with intact NHEJ, VDJ coding end-joining generates a diverse array of rejoined opened hairpin coding ends. In contrast, rejoining of the blunt signal ends is usually a precise, perfect head-to-head joining of the two heptamers. This recapitulates VDJ joining characteristics in developing lymphocytes. It has been shown that the rare VDJ joints generated in NHEJ deficient cells or animals have characteristics of a-EJ, such that coding joints have increased levels of nucleotide loss and the joints often occur at regions of MH; whereas signal ends are not perfectly rejoined. As shown above (Figure 2C), in cells completely deficient in Lig4, the joining rate of both coding and signal ends is severely reduced. Coding and signal joints from episomal assays were PCR amplified and analyzed by gel electrophoresis. As can be seen (Figure 8A, left), coding joints amplified from wild-type or Lig4+/− 293T cells generate a diverse ‘smear’ of coding joints, whereas coding joints amplified from 293T cells that express only catalytically inactive Lig4 are homogeneous, and slightly smaller than those recovered from wild-type cells.

As expected, signal joints from wild-type or Lig4+/− 293T cells are uniform, but signal ends isolated from cells that express catalytically inactive Lig4 are diverse, generating a smear of joints (Figure 8A middle). Rejoining of signal ends (without nucleotide loss or gain) generates a novel ApaLI site; so fidelity of signal end-joining can be ascertained by restricting signal joints with this enzyme. Signal joints amplified from assays in wild-type cells are uniform and largely susceptible to ApaLI cleavage consistent with perfect rejoining of signal joints in NHEJ-proficient cells (44). In contrast, signal joints amplified from cells that lack Lig4 or express only catalytically inactive Lig4 are completely resistant to ApaLI cleavage indicating nucleotide loss or addition from the signal ends prior to rejoining, suggesting catalytic inactive Lig4 complex is not proficient at promoting perfect blunt end-joining (Figure 8A, right).

PCR amplified coding joints were submitted for amplicon sequencing; as can be seen (Figure 8B), joints isolated from wild-type or Lig4+/− cells are virtually indistinguishable, and ∼50% do not have sequence MH at the site of joining. In these assays, there is a bias for joining at particular sites of microhomology resulting in over-representation of sequences with 3bp and 5bp deletions; these over-represented joints can be appreciated in all three samples. In wild-type cells, the predominant 3bp and 5bp deletion products account for 5.99% and 12.12% of all sequences, in Lig4+/− cells, 9.28% and 14.66%, but in K273A mutant cells these two joints account for 27.4% and 18.37% of all joints. These data support the conclusion that a higher fraction of joints facilitated by catalytically inactive Lig4 have characteristics of a-EJ. Still a large fraction of joints isolated from cells expressing the K273A mutant occur without the use of microhomology at the site of joining.

### Chromosomal end-joining in cells expressing K273A Lig4 has characteristics of a-EJ

**Figure 9.**
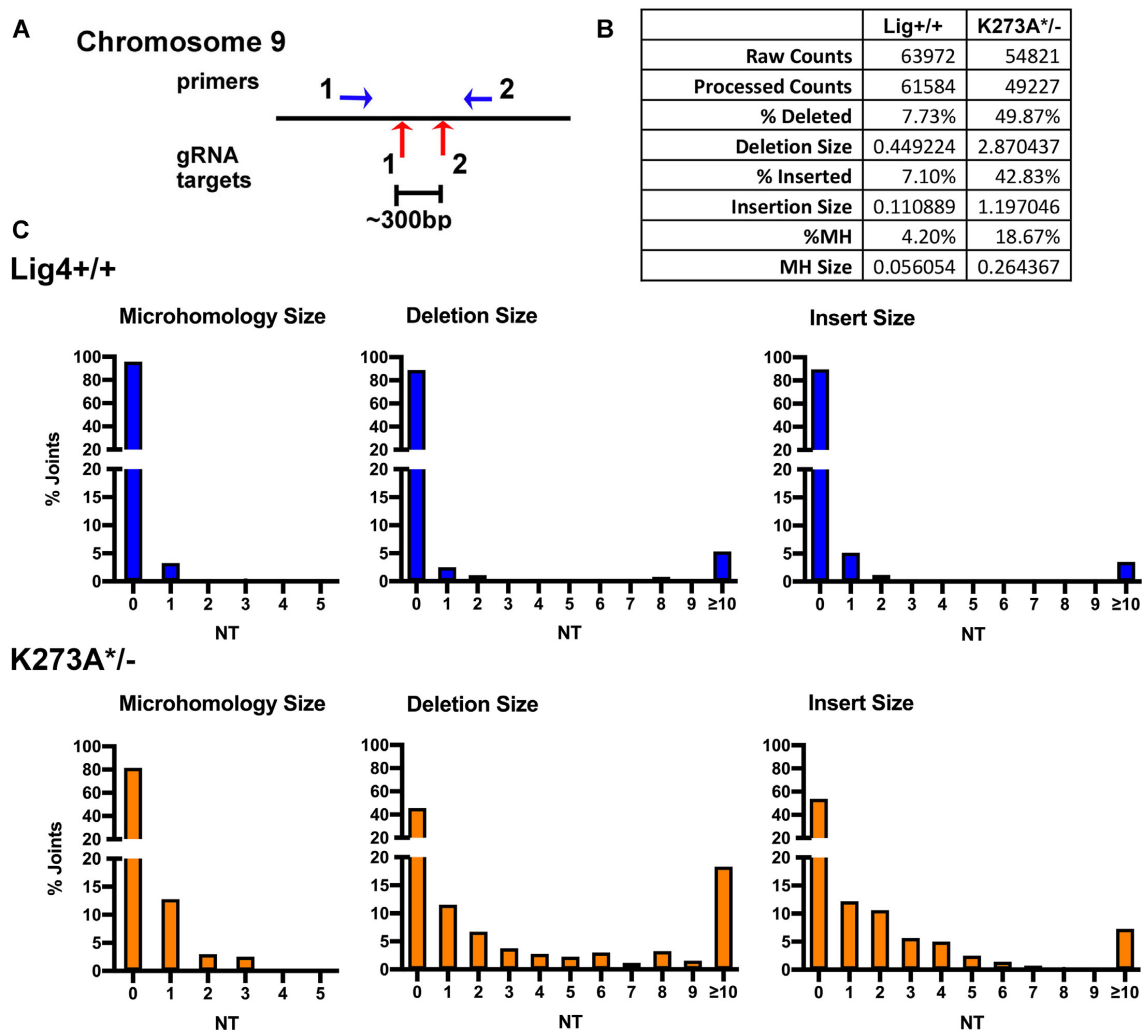
Chromosomal DSBs repaired in cells expressing K273A Lig4 have characteristics of a-EJ. **(A)** Diagram of region on chromosome nine targeted by two different gRNAs and position of primers utilized to detect chromosomal deletions induced by Cas9 and indicated gRNAs. **(B)** Summary of amplicon analyses of PCR amplified deletional joints from indicated cell strains. **(C)** Histograms depicting numbers of nucleotides deleted, inserted, or extent of microhomology at site of joining.

To extend these findings to chromosomal end-joining, we exploited CRISPR/Cas9 targeting of the FANCG gene on chromosome 9, which we found to be remarkably efficient in 293T cells (32). Although this strategy (Figure 9A) does not allow quantification of joining, the quality of joining can be assessed by sequencing. Briefly, cells of the indicated genotypes were transfected with two gRNA/cas9/puro plasmids that target sequences ∼300bp apart; after 48 hours, cells were subjected to puromycin selection. After 72 h, genomic DNA was isolated and PCR was utilized to assess deletional rejoining of the two DSBs. Isolated PCR fragments were submitted for amplicon sequencing. In wild-type cells, ∼92% of recovered joints are perfect (of the blunt-ended Cas9 cleavage sites), whereas in K273A mutant cells, only ∼80% of the joints are perfect (Figure 9B). In wild-type cells, the average nucleotide loss/joint is 0.45bp, and ∼4% of joints occur at sites of MH. In contrast, in K273A mutant cells, the average loss/joint was 2.9 bp, and ∼19% occur at sites with MH. The range of nucleotide loss, nucleotide insertion, and utilization of short sequence homologies for joints from wild-type or K273A mutant cells is shown in Figure 9C. We conclude that compared to joints isolated from wild-type cells, a larger fraction of joints isolated from K273A mutant cells have characteristics of a-EJ; still a high percentage of joints isolated from K273A mutant cells occur without the use of microhomology at the site of joining.

## DISCUSSION

In other mutational studies that block enzymatic function of NHEJ factors (i.e. Artemis or DNA-PKcs), inactivation results in a cellular phenotype that is similar to (45–47) or in certain cell types worse than complete loss (38,48). Thus, it appears that the Lig4 complex is unique in that in its inactive form, Lig4 can promote the function of other repair factors.

There have been several previous studies proposing various structural roles for the Lig4 complex. In 2007, Chu *et al.* reported that the NHEJ ligase complex promotes end-processing activities *in vitro* (11). This is consistent with more recent reports, where in an *in vitro* model of NHEJ, Stinson *et al.* demonstrated that Lig4 (either active or inactive) was necessary for formation of a short-range non-homologous end-joining complex that closely juxtaposed two DNA ends, promoting both end-processing and ligation (9).

Besides the studies from Loparro *et al.* suggesting a two-stage model of NHEJ (3,9), several other reports support a role in DNA end synapsis for the Lig4 complex (12–14). Reid *et al.* demonstrated that the Lig4 complex can promote end-synapsis *in vitro* (using purified proteins in a FRET assay), and the ability to synapse the ends was strongly impacted by DNA end structure, especially by the presence of the 5′ phosphate (13). Of note, a K273R mutant Lig4 complex was defective in synapsing DNA ends in their assays using purified proteins (13), whereas similar FRET assays from Graham *et al.* showed that K273A using *Xenopus* extracts, fully support end synapsis (3). Conlin *et al.*, extended the studies of end synapsis to show that a unique protrusion in Lig4 [first appreciated in structural studies (49)] allows synapsis of DNA ends with mis-matched termini (14). There are also cellular studies that support a role for the Lig4 complex in DNA end synapsis. Cottarel *et al.* demonstrated that cells deficient in Lig4 are defective in damage-induced autophosphorylation of DNA-PKcs at serine 2056 (S2056) (12). We first showed that S2056 autophosphorylation could occur in trans, and that S2056 phosphorylation occurs almost exclusively by autophosphorylation (50). Recent structural studies strongly support the conclusion that S2056 phosphorylation occurs in trans (24). Cottarel *et al.* demonstrated that catalytically inactive Lig4 could restore the deficit in S2056 phosphorylation in Lig4 deficient cells as well as wild-type Lig4. From these studies they proposed that the catalytically inactive Lig4 complex facilitates synapsis, promoting trans-autophosphorylation of DNA-PKcs at S2056 (12). We extended this finding by demonstrating that XLF also participates in the capacity of the Lig4 complex to promote S2056 phosphorylation in living cells, and that only XLF that was proficient in interacting with XRCC4 could promote S2056 phosphorylation (51).

Finally, Chiruvella *et al.* reported that a catalytically inactive Lig4 complex increases not only synapsis, but chromosomal end-joining in yeast; this impact on end-joining was absolutely dependent on XLF (15). This result is consistent with cellular assays presented here. However, unlike the impact we observe from the K273A mutant cells where joining can approach wild-type levels, in *Saccharomyces cerevisiae*, the levels of joining in strains expressing catalytically inactive Lig4 was still substantially lower than in wild-type cells. A potential explanation is that yeast lack Lig3 and joining in the catalytically inactive Lig4 mutant strains must be mediated by Lig1 (Cdc9 in yeast). Results presented in Figures 5 and 6 establish that *in vitro*, catalytically inactive human Lig4 strongly stimulates Lig3 (but marginally Lig1) activity. However, previous studies in mammalian cells have shown that either Lig1 or Lig3 can function to promote a-EJ in Lig4 deficient cells (31,52,53) and that a-EJ still occurs in Lig3, Lig4 proficient cells (41). Thus, it is possible that Lig1 may also contribute to the joining observed in mammalian cells expressing catalytically inactive Lig4.

Our working model is that Lig4 itself is important in promoting synapsis and that formation of a short-range synaptic complex (even one that lacks Lig4 activity): 1) facilitates joining of many DSBs, 2) promotes cellular resistance to drugs that induce DSBs in some cell types, and 3) fulfills the RAG-induced restriction step of VDJ recombination so that even RAG-induced DSBs (both coding and signal ends) can be joined by non-NHEJ DNA ligases.

In sum, data presented here extend these previous studies demonstrating a non-catalytic role for Lig4 that robustly stimulates both end-joining and cellular resistance to DNA damage. Joining that is facilitated by the inactive Lig4 complex shares characteristics with a-EJ (more joints with increased nucleotide loss, and increased use of microhomologies), although a substantial fraction of joints occur without MH at the site of joining. This observation presents an unanticipated possibility, that the inactive Lig4 complex (likely consistent with short-range complexes proposed recently) may facilitate a-EJ mediated by Lig3. These studies present an important unanswered question: Do complexes with catalytically active Lig4 utilize Lig3 to facilitate joining in normal cells? Work is ongoing to address this possibility.

## DATA AVAILABILITY

The authors confirm that the data supporting the findings of this study are available within the article and its supplementary materials; the amplicon sequencing files from this study are available from the corresponding author.

## Supplementary Material

gkac913_Supplemental_File

## Notes

Present address: Weifeng Mao, Department of Biotechnology, Dalian Medical University, Dalian 116044, China.
