# Supplementary material for: Catalytically inactive DNA ligase IV promotes DNA repair in living cells
Source: Nucleic Acids Res. 2022 Oct 20;50(19):11058–71. doi: 10.1093/nar/gkac913 (PMC9638927; doi:10.1093/nar/gkac913)
Supplement: gkac913_Supplemental_File [file gkac913_supplemental_file.pdf]

Supplementary Data

Supplementary Table 1.

**L4 Wild-type**	pam<br>269 | 273<br>TAC ATA GAA ACC AAG CTA GAT GGT GAA<br>Y I E N K L D G E	
**HDR oligo**	TAC ATA GAA ACA GCG CTA GAT GGT GAA<br>Y I E N A L D G E	
**Clone 144**<br>**1st allele**	TAC ATA GAA ACC AAG C-- --- GGT GAA<br>Y I E N K FS stop	50% of amplicon reads
**2nd allele**	TAC ATA GAA ACA GCG CTA GAT GGT GAA<br>Y I E N A L D G E	50% of amplicon reads
**Clone 195**<br>**1st allele**	TAC ATA GAA ACC AAG CTT AGA TGG TGA A<br>Y I E N K L R W stop	52% of amplicon reads
**2nd allele**	TAT GTA GAA ACA GCG CTA GAT GGT GAA<br>Y V E N A L D G E	48% of amplicon reads
**Clone 17**<br>**1st allele**	TAC ATA GAA ACC AAG CT- --- GGT GAA<br>Y I E N K L FS	Sanger sequence
**2nd allele**	TA- --- --- --- --- --- GAT GGT GAA<br>FS stop	Sanger sequence
**Clone 104**<br>**1st allele**	TAC ATA GAA ACC AAG CGC TAG ATG GTG<br>Y I E N K L stop	47% amplicon
**2nd allele**	TAC ATA GAA ACC AAG CTA GAT GGT GAA<br>Y I E N K L D G E	47% amplicon

Supplementary Table 1. Mutant and wild type Lig4 alleles in 293T clones.

Supplementary Figure 1.

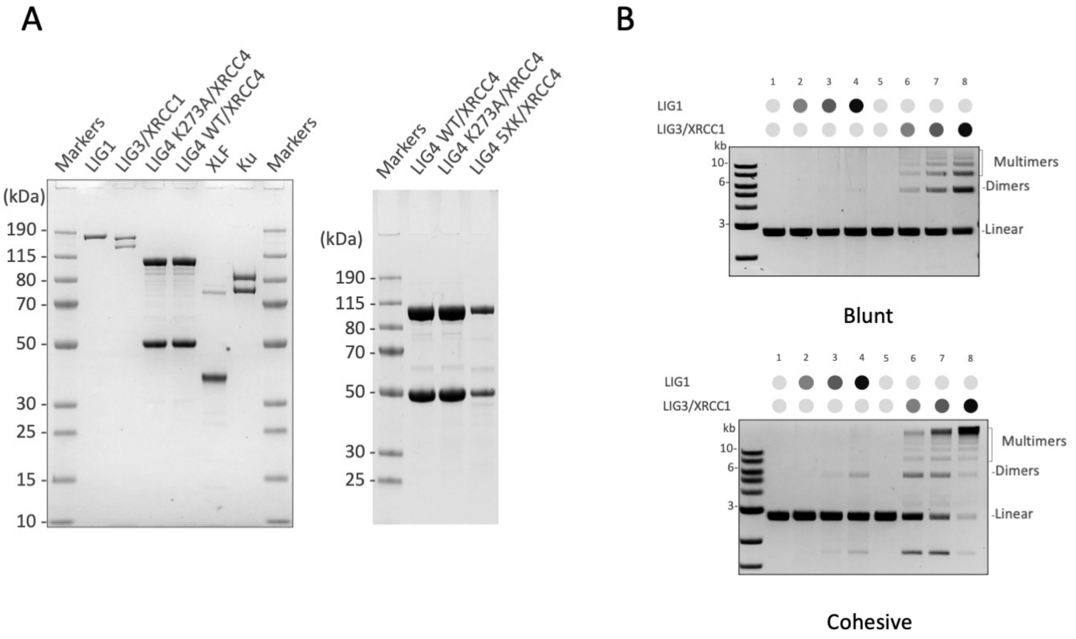

Supplementary Figure 1. Purified NHEJ proteins and ligase activity assays for Lig1 and Lig3/XRCC1. (A) Coomassie gel of purified NHEJ factors Ku, XLF, Lig4-WT/XRCC4, Lig4-K273A/XRCC4, and Lig4-5XK/XRCC4 as well as Lig1 and Lig3/XRCC1 (note Lig1, Lig3 and XRCC1 are N-terminally tagged with MBP). **(B)** Lig1 and Lig3/XRCC1 DNA ligation assays using a linear DNA fragment (2.7 kb) with blunt or cohesive ends (Lig1 was added at 0, 100, 200, and 300 nM; and Lig3/XRCC1 at 0, 50, 100, and 150 nM final concentrations). Reactions (10 μL) containing 50 ng of linear pUC19 were incubated for 30 minutes at room temperature and analyzed by agarose gel electrophoresis after Proteinase K treatment.

Supplementary Figure 2.

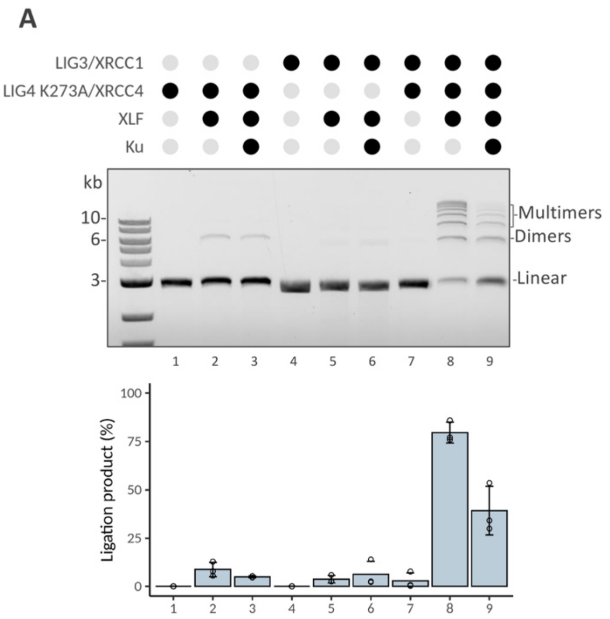

Supplementary Figure 2. K273A Lig4/XRCC4/XLF retains residual joining activity but promotes robust Lig3-mediated joining of cohesive DNA ends *in vitro.* The capacity for catalytically inactive Lig4/XRCC4 to promote ligation by Lig3 **(A)**, or Lig1 **(B)** was assessed by incubating a cohesive DNA ligation substrate (XbaI linearized pUC19) with purified recombinant proteins as indicated at the following concentrations: Ku, 250 nM; XLF, 200 nM; Lig4/XRCC4, 500 nM; Lig3/XRCC1, 40 nM; and Lig1,180 nM. Reactions (10 µl) were incubated for 30 minutes at room temperature, deproteinized and products resolved by agarose gel electrophoresis before detection and quantification with Image J. Bar graph represents three independent experiments and error bars represent SD.

Supplementary Figure 3.

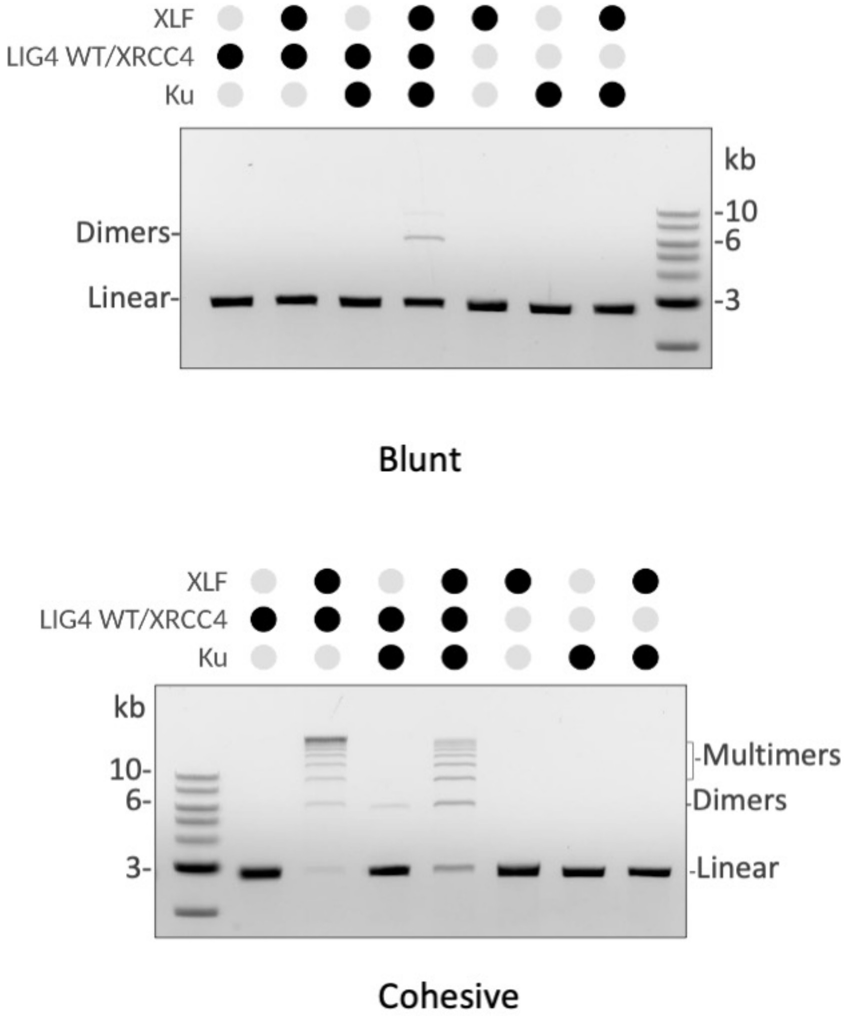

Supplementary Figure 3. Purified NHEJ factors support ligation of both blunt and cohesive DNA ends *in vitro*. DNA ligation assays using a linear DNA fragment (2.7 kb) with blunt or cohesive ends. Reactions (10 µL) containing 50 ng of linear pUC19 and the following final protein concentrations: Ku, 250 nM; XLF, 200 nM; Lig4 WT/XRCC4, 500 nM. were incubated for 30 minutes at room temperature, treated Proteinase K and resolved by agarose gel electrophoresis prior to detection

Supplementary Figure 4.

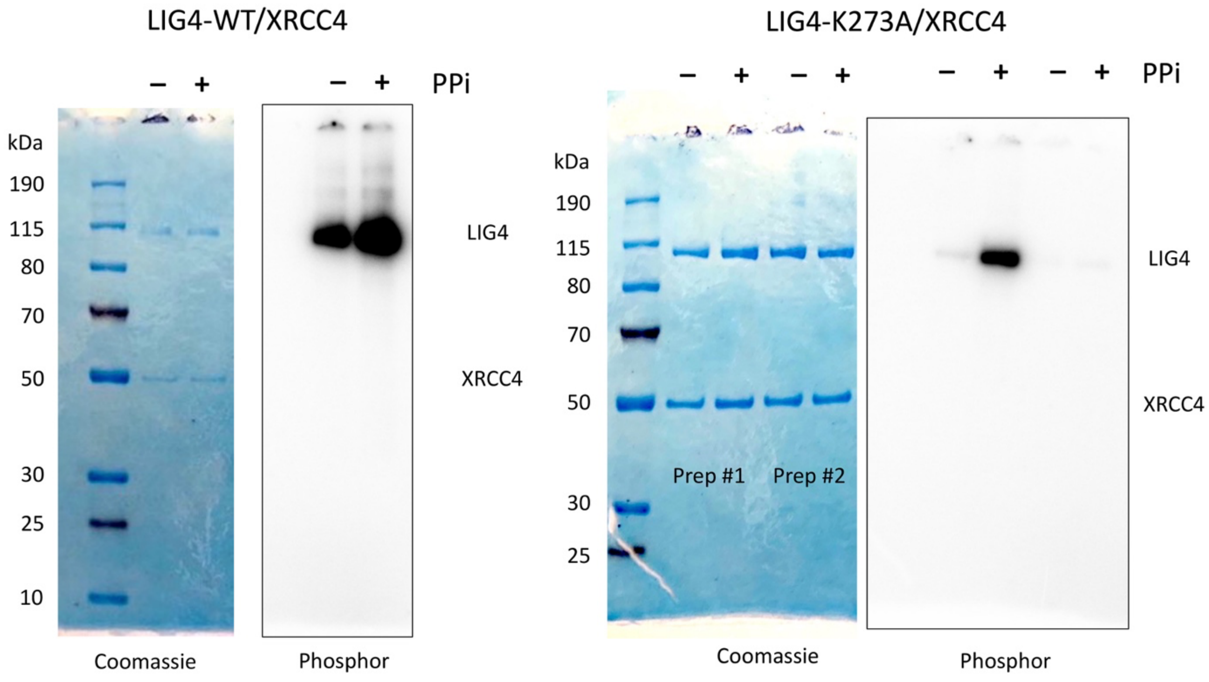

Supplementary Figure 4. K273A mutant Lig4 is weakly adenylated. Lig4-WT/XRCC4 (5 μM) and two different preparations of Lig4-K273A/XRCC4 (10 μM) were incubated with α ^{32}P ATP with or without first incubating with pyrophosphate. Dried Coomassie stained gels were analyzed on a phosphorimager for radioactive label detection. Level of K273A adenylation varies in different protein preparations but correlates with residual ligase activity (not shown). Mass spectrometric analyses confirmed that the Lig4 K273A/XRCC4 preparations contain uniformly the K273A mutation in the Lig4 protein (data not shown).
